# Admission Clerking- Inpatient Adult Psychiatric Unit - a Quality Improvement Project

**DOI:** 10.1192/bjo.2022.288

**Published:** 2022-06-20

**Authors:** Asha Dhandapani, Sathyan Soundara Rajan, Alberto Salmoiraghi, Rajvinder Sambhi, Catherine Baker, Joanne Kendrick, Sherrie Stewart

**Affiliations:** Betsi Cadwaladr University Health Board, Wrexham, United Kingdom

## Abstract

**Aims:**

To improve the clerking proforma and physical healthcare for General Adult Psychiatric inpatients in Heddfan Psychiatric Unit, Wrexham by 100% within 18 months period with a long term goal of continuous improvement.

**Methods:**

We started the project with a baseline audit which showed the incompleteness of vital data when clerking a patient in adult psychiatric inpatient unit. This was compared with various standards from Core competencies for a trainee in Psychiatry, NICE guidelines and Local trust policy from our own trust BCUHB for physical health monitoring and Department of Health Guideline for VTE.

With the findings obtained, we went ahead to create a proforma encompassing all the details.

The use of various Quality improvement tools such as Fishbone diagram, Drivers diagram and PDSA cycles gave as overwhelming results

**Results:**

The baseline audit, repeat audits and PDSA cycles have shown tremendous and overwhelming results in terms of completion of the proforma. This has resulted in mandatory details being inputted sufficiently in the patient's notes.

Many of the important details such as medication details, allergy status, legal and forensic status, mental state examination, risk assessment, VTE assessment, investigation details and documentation have shown to have improved during this 1 year

**Conclusion:**

This QIP has been patient centred as this is the main goal. Following the PDSA cycle, we have identified that it has been efficient and effective. It has been safe and also reduced the chances of patient neglect. The structure of the proforma used does not discriminate or show any inequalities and is timesaving too.

The SWOT analysis has been completed, which has shown that the teamwork and support from the Consultants and other stakeholders have been a major strength. There are a few weaknesses such as unavailability of ECG machine, missing documentation of investigations despite completing them but however with timely education to the junior doctors, we are hoping for improvement further. This QIP has opened up doors for various opportunities, such as including nursing and pharmacy admission forms into this proforma. Though there are few threats in achieving 100% success, we are hoping for the best

